# Enhancing the Potential of Immunotherapy in Paediatric Sarcomas: Breaking the Immunosuppressive Barrier with Receptor Tyrosine Kinase Inhibitors

**DOI:** 10.3390/biomedicines9121798

**Published:** 2021-11-30

**Authors:** Emmy D. G. Fleuren, Rachael L. Terry, Deborah Meyran, Natacha Omer, Joseph A. Trapani, Michelle Haber, Paul J. Neeson, Paul G. Ekert

**Affiliations:** 1 Children’s Cancer Institute, Lowy Cancer Research Centre, UNSW Sydney, Randwick 2031, Australia; rterry@ccia.org.au (R.L.T.); mhaber@ccia.unsw.edu.au (M.H.); pekert@ccia.org.au (P.G.E.); 2 School of Women’s and Children’s Health, UNSW Sydney, Randwick 2052, Australia; 3 Centre for Childhood Cancer Research, UNSW Sydney, Randwick 2031, Australia; 4 Cancer Immunology Program, Peter MacCallum Cancer Centre, Melbourne 3000, Australia; Deborah.Meyran@petermac.org (D.M.); joe.trapani@petermac.org (J.A.T.); paul.neeson@petermac.org (P.J.N.); 5 Sir Peter MacCallum Department of Oncology, University of Melbourne, Melbourne 3000, Australia; 6 Inserm, Université de Paris, U976 HIPI Unit, Institut de Recherche Saint-Louis, 75475 Paris, France; 7 Translational Innate Immunotherapy, University of Queensland Diamantina Institute (UQDI), Brisbane 4102, Australia; Natacha.Omer@health.qld.gov.au; 8 Oncology Services Group, Queensland Children’s Hospital, Brisbane 4101, Australia; 9 Murdoch Children’s Research Institute, Royal Children’s Hospital, Melbourne 3052, Australia

**Keywords:** paediatric and AYA sarcoma, immunotherapy, immune checkpoint inhibitors, receptor tyrosine kinase inhibitors

## Abstract

Despite aggressive surgery, chemotherapy, and radiotherapy, survival of children and adolescents and young adults (AYAs) with sarcoma has not improved significantly in the past four decades. Immune checkpoint inhibitors (ICIs) are an exciting type of immunotherapy that offer new opportunities for the treatment of paediatric and AYA sarcomas. However, to date, most children do not derive a benefit from this type of treatment as a monotherapy. The immunosuppressive tumour microenvironment is a major barrier limiting their efficacy. Combinations of ICIs, such as anti-PD-1 therapy, with targeted molecular therapies that have immunomodulatory properties may be the key to breaking through immunosuppressive barriers and improving patient outcomes. Preclinical studies have indicated that several receptor tyrosine kinase inhibitors (RTKi) can alter the tumour microenvironment and boost the efficacy of anti-PD-1 therapy. A number of these combinations have entered phase-1/2 clinical trials, mostly in adults, and in most instances have shown efficacy with manageable side-effects. In this review, we discuss the status of ICI therapy in paediatric and AYA sarcomas and the rationale for co-treatment with RTKis. We highlight new opportunities for the integration of ICI therapy with RTK inhibitors, to improve outcomes for children with sarcoma.

## 1. Introduction

Sarcomas are an incredibly heterogeneous group of mesenchymal tumours with >150 different histological entities recognised. Sarcomas account for >10% of childhood cancers [1]. The most common sarcomas that occur in children and adolescents and young adults (AYAs) are the bone sarcomas, osteosarcoma and Ewing sarcoma, and rhabdomyosarcoma (RMS), a soft-tissue sarcoma (STS). Other, less commonly observed STS subtypes associated with a younger age of onset are the malignant peripheral nerve sheath tumours (MPNST), synovial sarcomas (SS), alveolar soft part sarcomas (ASPS), and desmoplastic small round cell tumours (DSRCT) [2,3]. For virtually all these tumours, a plateau has been reached in the efficacy of current treatment approaches, with few significant developments in the treatment of these bone and soft tissue tumours in decades [4]. Children with recurrent, relapsed, or metastatic disease only have a 20–30% chance of survival, despite intensive chemotherapy and radiotherapy [5]. No targeted therapy is approved for routine clinical use in these young patients.

The field of immunotherapy is a rapidly evolving discipline, aimed at strategically invoking an endogenous immune response to target and kill tumour cells. Immune checkpoint inhibitors (ICIs) are an exciting type of immunotherapy that release T cells and natural-killer (NK) cells from tumour-mediated immunosuppression, allowing them to kill tumour cells [6]. The most heavily investigated ICIs are antibodies directed against the immune checkpoints programmed death-1 (PD-1) and its ligand programmed death ligand-1 (PD-L1), as well as cytotoxic T-lymphocyte-associated protein 4 (CTLA-4) [7]. PD-1-targeted therapies have achieved remarkable results in several adult cancers, particularly in melanoma and non-small-cell lung cancer [8]. In the handful of studies testing these agents in paediatric sarcomas, limited efficacy was observed. However, individual responses to single agent ICIs have been reported across different paediatric sarcoma subtypes, with outcomes ranging from stable disease (SD) and partial response (PR), to even a complete response in a patient with Ewing sarcoma [9,10,11]. This illustrates that these inhibitors may be beneficial in selected paediatric sarcomas and provides a rationale to explore therapeutic combinations that may enhance their efficacy in a larger group of paediatric sarcomas that currently do not derive meaningful clinical benefit from these agents as monotherapies. 

One major barrier to the efficacy of ICIs is the intrinsically immunosuppressive nature of the tumour microenvironment (TME). The crux lies in identifying innovative ways to break through these immunosuppressive barriers. Interestingly, there is a growing body of data to show that a number of receptor tyrosine kinase inhibitors (RTKis), many of which are currently being trialled for paediatric sarcoma management, have immunomodulatory effects on the TME through depletion or reprogramming of immunosuppressive cell subsets and enhancement of T cell infiltration. A combination of immune checkpoint inhibitors with these RTKis could hence fulfil the promise of both these classes of drugs by significantly improving outcomes for young sarcoma patients. 

Here, we outline the status of ICI therapy in paediatric sarcomas and discuss their overall limited efficacy as a monotherapy in this group of patients [9]. We review the RTKis that are currently in trials for children and AYAs with sarcoma, with a particular focus on those with immunomodulatory effects on the TME. Finally, we bring these approaches together and highlight the rationale for combining ICIs with RTKis, report early safety and efficacy data on these combinations in paediatric and AYA sarcoma patients, and highlight a number of successful adult sarcoma trials in this field to help pinpoint the most promising ICI and RTKi combinations that should be considered for future paediatric and AYA sarcoma clinical trials.

## 2. Immune Checkpoint Inhibitors in Paediatric Sarcoma

There have been several trials investigating the efficacy of ICIs, mainly utilising antibodies directed against PD-1, in paediatric and AYA sarcoma patients. The first trial testing the anti-PD-1 antibody pembrolizumab in children and adults with sarcoma was published in 2017 [12]. Although only adult patients with STS subtypes (>18 year) were included, patients from the age of 12 were eligible to be enrolled in the bone sarcoma cohort. Within this cohort, one PR and six SDs were reported among 22 osteosarcoma patients, resulting in an objective response rate (ORR; combined CR and PR) of 5% and a clinical benefit rate (CBR; combined CR, PR, and SD) of 32%. From the 13 enrolled Ewing sarcoma patients, two had SD (CBR: 15%). Of the ten patients included with SS, one PR and two SDs were reported (ORR: 10%; CBR: 30%). A phase-2 trial investigating the efficacy of pembrolizumab in advanced osteosarcoma reported SD in 4 out of 12 osteosarcoma patients as the best response. However, none of the patients achieved the primary endpoint of CR, PR, or SD at 18 weeks, and hence it was concluded that pembrolizumab had no meaningful clinical activity in osteosarcoma patients [13]. Retrospective analyses of individual responders from different institutes reported antitumour efficacy of the anti-PD-1 antibody nivolumab in a 12-year-old patient with metastatic osteosarcoma and a 16-year-old with metastatic Ewing sarcoma [14]. Objective responses of pembrolizumab were also reported in 5 out of 12 adult STS patients in another study (ORR: 42%), although no response was reported in the sole Ewing sarcoma patient [15]. In a retrospective analysis published in 2020, responses to pembrolizumab were examined in 14 evaluable AYA patients with advanced bone and soft tissue sarcomas (ORR: 14%; CBR: 21%) [11]. One of the most profound responses was observed in one out of the three evaluable Ewing sarcoma patients, who showed a CR. The 25-year-old patient stopped treatment after nine cycles and remained in remission for at least 48 months. Because this patient had multiple sites irradiated, the authors speculated that this may have resulted in a more immunogenic state of the tumour, which contributed to the complete sustained response [11]. Radiotherapy, chemotherapy, and certain targeted therapies have all shown potential to transform tumours to a more immunogenic state, albeit via different mechanisms. The premise for all these treatments is, however, very similar: enhancement of the effects of immunotherapy when given in combination [16,17]. The profound effect observed in the Ewing sarcoma patient hence further underpins our rationale of combining ICIs with immunomodulating drugs to enhance ICI efficacy, also in young sarcoma patients. Two of the three patients with ASPS, both in their twenties, also showed a clinical benefit from pembrolizumab in the same study, where one achieved a PR that lasted at least 2 years, and the other showed SD. The included four osteosarcoma, three SS, and one clear-cell sarcoma patients all showed progressive disease [11].

In a phase-1/2 trial published in 2020, children and young adults with relapsed/refractory solid tumours, including patients with RMS, Ewing sarcoma, and osteosarcoma, were treated with nivolumab [18]. Although there were no objective responders among the sarcoma patients in this study, SD was reported in a minority of patients in each sarcoma cohort. Finally, another study from 2020 tested the efficacy of pembrolizumab in paediatric patients with advanced or relapsed solid tumours [19]. Of 136 patients with solid tumours, 8 patients showed a PR, which included one patient with an epithelioid sarcoma. Altogether, these studies show that anti-PD-1 has limited efficacy as a monotherapy in paediatric sarcoma, even though the occasional individual responses hint that in selected instances, ICI therapy can be beneficial. This raises questions about the selection of sarcoma patients for ICI therapy and about the optimal application of this treatment. 

## 3. Biomarkers of Immune Checkpoint Inhibitor Therapy Response?

An important critique to the limited efficacy of anti-PD-1 monotherapy in paediatric sarcoma patients is that many trials, particularly the earlier ones, did not include *a priori* testing for a predictive biomarker of response, such as immunohistochemical assessment of PD-1/PD-L1 tumour expression, to select patients most likely to respond. Although the expression of PD-L1 has been reported to be present in between 47–86% of paediatric sarcoma subtypes [20], there is significant variability in reported PD-L1 expression levels within different sarcoma subtypes, in the disease stage of sampling, the PD-L1 antibody clone used, the methods of tissue preservation employed, the subjective interpretation of immunohistochemical results, and the limited sample size [21,22,23]. It does, hence, not come as a surprise that the role between PD-1/PD-L1 expression and response to PD-1 inhibitors is inconsistent, and responses to PD-L1 therapy have been reported in apparently PD-L1-negative sarcomas [12]. 

The inconsistent predictive value of PD-1/PD-L1 expression has prompted further research into other biomarkers that may be more accurately reflecting predictions to ICI therapy responsiveness, by providing more insights into the complexity of the TME. These include a number of tumour-intrinsic biomarkers, including tumour mutational burden (TMB), the presence of neoantigens, the microsatellite instability (MSI) status, DNA damage repair defects, and the interferon-γ gene signature [24]. Together with more immune-specific biomarkers such as T cell infiltration, the B cell gene-expression signature, or tertiary lymphoid structure, they each correlate with response to ICIs [25,26,27]. However, these single biomarker strategies do not incorporate the complex and dynamic interaction of the tumour and host immune system, and combinatorial biomarkers, bridging clinical investigations with preclinical mechanistic studies, are needed to optimize patient selection [28]. A recent study of more than 1000 ICI-treated patients across seven tumour types showed that a multivariable predictor of response outperformed TMB alone [29]. Interestingly, the presence of mature tertiary lymphoid structures (TLSs) was recently shown to be associated with improved objective response rates, progression-free survival, and overall survival in a retrospective analysis of three independent cohorts of patients with cancer who were treated with anti-PD-1 or anti-PD-L1 antibodies, independent of PD-L1 expression status and CD8^+^ T cell density [30].

In the SARC028 trial, which investigated the efficacy of pembrolizumab in advanced sarcoma, PD-L1 positivity, infiltrating lymphocyte density, and the abundance of tumour-infiltrating macrophages correlated with the response to monotherapy in undifferentiated pleomorphic sarcoma and dedifferentiated pleomorphic liposarcoma [31]. A review of immunohistochemistry-based immune biomarker studies in sarcomas, which included PD-L1, FoxP3, and CD8, suggested a positive association with improved survival in specific histologic subtypes, for example, PD-L1 in alveolar RMS, CD163 in embryonal RMS and CD8 in SS [32]. However, beyond individual cases, ICIs have been ineffective in these subtypes, and no consensus of the predictive value of these biomarkers can be reached. A more tiered assessment of the TME may have a better predictive value. Petitprez et al. classified sarcomas into five categories: immune-low (A and B), vascular (C), and immune-“hot” (D and E) [27]. The immune-high E category, which is particularly rich in B cells, demonstrated a high response rate and improved survival to ICIs.

Overall, tumour-intrinsic biomarkers (high TMB, high neoantigen load, and MSI-high status) are rare in sarcomas, particularly in those sarcoma subtypes affecting the young, and immune-specific biomarkers are very dependent on the sarcoma histological subtypes. It was demonstrated that “hot” sarcomas with increased immune infiltrates are more likely to respond to ICIs, yet paediatric sarcomas are in most instances classified as “cold”. Since the immunogenic state of a tumour has a major influence on the efficacy of ICIs, a rational therapeutic approach is to break down the immunosuppressive barrier with combination therapies and to transform “cold” paediatric sarcomas to a more immunogenic “hot” phenotype [4].

## 4. Receptor Tyrosine Kinase Inhibitors

Receptor tyrosine kinases (RTKs) are membrane-bound proteins that are critical mediators regulating cell growth, proliferation, and survival [33]. As they play a pivotal role in normal physiology, they are present on the surface of many different cells. In most normal cells, including immune cells, RTKs are normally expressed at low levels. Overexpression and activation of RTKs is a feature of many cancer types, including paediatric and AYA sarcomas, even in the absence of activating mutations or structural variants [34,35,36]. Consequently, several RTK inhibitors have been tested in the preclinical and clinical setting in these tumours, to inhibit potentially oncogenic RTK signalling within the tumour cells [36,37]. 

The RTKis that have had most clinical success in paediatric sarcomas to date include multi-RTK inhibitors with largely overlapping profiles of molecular inhibition. They all combine a strong anti-angiogenic component, via direct inhibition of the vascular endothelial growth factor receptors (VEGFRs), with the inhibition of key oncogenic RTKs implicated in paediatric and AYA sarcoma, including the platelet-derived growth factor receptors (PDGFRs), c-KIT, fibroblast growth factor receptors (FGFRs), RET, and/or MET. Sorafenib [38], regorafenib [39,40], lenvatinib [41], and anlotinib [42], all of which have activity against VEGFR, PDGFR, RET, c-KIT and FGFR, and cabozantinib [43], which has a different target profile (VEGFR, MET, AXL, and RET), have all shown some clinical benefit in sarcoma. The response rates (any response of SD, PR, or CR response) varies from 60–100% in osteosarcoma and Ewing sarcoma patients, and there are modest gains in progression-free survival (PFS) of between 3.6 and 6.7 months with these single agents. The clinical responses and PFS improved even further when these drugs were combined with chemotherapy, underlining the rationale for combination therapies for these types of inhibitors.

The most pronounced effects in osteosarcoma and Ewing sarcoma were reported with anlotinib, cabozantinib, and lenvatinib. Anlotinib monotherapy resulted in a median PFS of 5.3 months in a cohort of children and adults with advanced bone sarcoma patients, consisting of 29 osteosarcoma and 3 Ewing sarcoma patients out of 42 bone sarcoma patients in total, with clinical benefits reported in 79% of these patients [42]. With cabozantinib monotherapy, PR and SD were reported as the best overall response in 7 and 26 out of 41 evaluable childhood and adult osteosarcoma patients, respectively. This equated to a CBR of 80% and a median PFS of 6.7 months. In the same trial, cabozantinib was also very effective for Ewing sarcomas, where 10 patients achieved a PR and 19 patients SD from 37 evaluable Ewing sarcoma patients, equating to a CBR of 78% and a median PFS of 4.4 months [43]. With lenvatinib monotherapy, disease control and ORR were achieved in 53% and 7% of young patients with relapsed or refractory osteosarcoma, respectively [44]. Several studies demonstrated the increased benefit when RTK-targeted drugs were combined with chemotherapeutic agents, where particularly the combination of lenvatinib with etoposide and ifosfamide appeared remarkably effective in the clinic. This combination very recently showed an impressive median PFS and overall survival (OS) of 8.7 and 16.3 months respectively, for heavily pre-treated osteosarcoma patients between 2 and 25 years, with manageable side effects [41]. These results are incredibly encouraging, as all trials were performed on a population of heavily pre-treated patients that had exhausted all other treatment options. The expected median PFS for osteosarcoma patients at this stage, if left untreated, is between 1–1.8 months [39,40]. The toxicity profiles of these drugs are manageable. As extension of survival was only observed in patients treated with an RTKi combined with chemotherapy, but not in any of the monotherapy trials, this underlines the importance of designing rational, biologically based combination therapies to maximise the efficacy of RTK-directed strategies. The question remains, apart from standard-of-care chemotherapy, which type of combination therapy would work best for these inhibitors in this population. A clue to the answer to this key question can be found when considering the biological effects that RTKis exert on a tumour. In addition to directly inhibiting their target molecules on the surface of the tumour cells, they also affect the tumour microenvironment. RTKis have been shown to be capable of inactivating or normalising essentially the complete range of deregulated TME components, including endothelial cells, (cancer-associated) fibroblasts, stem cells, and even non-cellular components. Their potent modulatory effects on different types of immune cells, particularly on immunosuppressive cells such as myeloid-derived suppressor cells (MDSCs), tumour-associated macrophages (TAMs), and regulatory T cells make RTKis of particular interest in terms of combination strategies with immunotherapy [45].

## 5. Immunomodulatory Effects of Receptor Tyrosine Kinase Inhibitors

The immune microenvironment of solid tumours is highly immunosuppressive [46]. The tumour microenvironment is populated with several immune cell populations that inhibit T cell responses against the tumour, including MDSCs, TAMs, and regulatory T cells [47]. These cells suppress anti-tumour T cell activity through a variety of mechanisms such as the expression of immune checkpoint molecules, the production of anti-inflammatory cytokines such as IL-10 and transforming growth factor beta (TGF-ß), and the restriction of metabolites, such as tryptophan, that are critical for T cell proliferation, activation, and function [46,47].

There is a growing body of data to show that RTKis have immunomodulatory effects on the TME, through depletion or reprogramming of immunosuppressive cell subsets and enhancement of T cell infiltration (Table 1) [45]. Interestingly, the RTKis that have shown these immunomodulatory effects are the same RTKis that have shown the most promising clinical effects in paediatric sarcoma [41,42,43]. There is evidence suggesting that for some RTKis, these effects are, at least in part, mediated through direct suppression of immune-suppressing cells [48]. Cabozantinib, for example, decreased the number and function of MDSCs in preclinical prostate cancer mouse models, where in vitro studies confirmed a direct suppressive effect on MDSCs, thereby blocking their suppressive activity on CD4^+^ and CD8^+^ T cell proliferation. Although cabozantinib alone, or a cocktail of ICIs alone, had minimal impact on the mass of these prostate tumours, the combination of the two was potently synergistic in targeting primary and metastatic prostate cancer growth. This was largely attributed to the effects of cabozantinib on the TME [48]. Additionally, in models of other cancer types, cabozantinib was shown to effectively target both the adaptive and innate immune system, resulting in synergistic effects when combined with either ICIs or a cancer vaccine [49]. Interestingly, despite the fact that the effect of RTKis on immune cell activation and neutrophils in particular has only rarely been described, another study in murine prostate cancer models showed that cabozantinib treatment was associated with enhanced release of neutrophil chemotactic factors, resulting in robust neutrophil infiltration into the prostate tumours [50].

Lenvatinib also showed promising immunomodulatory effects in preclinical cancer models. In a hepatocellular carcinoma model in immunodeficient mice, lenvatinib and another tyrosine kinase inhibitor, sorafenib, had similar anti-tumour efficacies. However, in immunocompetent mice, lenvatinib was more potent than sorafenib. One explanation was that lenvatinib treatment decreased the proportion of monocytes and macrophages in the tumour and increased the CD8^+^ T cell infiltration [51]. In terms of activating immunomodulating properties, in murine melanoma and renal cancer models, lenvatinib was further shown to enhance the tumor infiltration and activation of NK cells [52]. These findings suggest that the activity of lenvatinib includes important immunomodulatory activity and that combination with immunomodulating agents, such as an anti-PD-1 antibody, may enhance anti-tumour efficacy.

## 6. Combining ICIs with RTKis in Paediatric and AYA Sarcomas

It is evident that for both RTK-directed therapies and ICI-directed therapies, single-agent treatment strategies are insufficient to cure paediatric and AYA sarcoma patients. The increasingly recognised immunomodulating properties of RTKis, together with their ongoing clinical use in paediatric sarcoma patients, makes the combination of RTKis with ICIs a rational and attractive way forward for treating paediatric sarcomas and could fulfil the promise of deriving meaningful clinical responses for these young patients. These combinations are currently being tested in clinical trials in a wide range of adult cancers, including several sarcoma-specific trials. A smaller number of studies include younger patients (Table 2 and Table 3).

A phase-2 trial investigating the combination of the VEGFR2 inhibitor apatinib and the PD-1 inhibitor camrelizumab in osteosarcoma patients of 11 years old or older reported clinical benefits in 30% of patients at 6 months, an ORR in 21% (9 out of 43 patients), and a median PFS of 6.2 months [60]. Treatments were administered semi-simultaneously, where apatinib was taken orally each day, and camrelizumab was administered intravenously every two weeks in a four-week cycle. The number of objective responses is impressive and unmatched by single-agent PD-1 inhibitors in this tumour type. Although this combination also prolonged PFS compared to single-agent apatinib, which is reported to be 4.5 months [61], and this combination was particularly effective in osteosarcoma patients with pulmonary metastatic lesions, it did not reach the overall prespecified endpoint of 6-month PFS of 60% or greater [60]. Interestingly, as osteosarcoma patients with pulmonary metastases and patients with overexpression of PD-L1 had a significantly longer PFS compared to other patients, this points towards the possibility and importance of stratification of osteosarcoma patients. The use of more-refined markers, such as immune-response biomarkers, in future clinical trials is expected to pave the road towards a more comprehensive understanding on the biology of responding and non-responding tumours [60]. Of note, other RTKi-plus-PD-1 inhibition combination trials have less-stringent study primary endpoints (15–30% of patients free from progression at 6 months) [62,63]. For example, the combination of sunitinib (VEGFR/PDGFR/RET/c-KIT multi-RTK inhibitor) and nivolumab in bone sarcomas and advanced STS patients showed progression-free survival in 32% and 48% of patients, respectively, at 6 months, thereby meeting the primary study endpoints of >30% and >15% PFS [62,63]. For the STS cohort, based on these endpoints, sunitinib plus nivolumab was concluded to be an active combination with manageable toxicity with almost half of patients free of progression at 6 months [63]. One of two patients with SS showed a PR to this combination [63], and in another study, the one enrolled SS patient showed a minor response to the combination of pembrolizumab and the VEGFR-inhibitor axitinib [64]. In the bone sarcoma cohort of the sunitinib and nivolumab trial, marked efficacy was observed in heavily pre-treated patients with a CBR of 60% (24 out of 40 patients). A CR was reported in 1 out of 14 chondrosarcoma patients (lasting over 22 months), PR in 1 out of 17 osteosarcoma patients (lasting 5.7 months), and SD in 22 not-further-specified bone sarcoma patients (lasting > 6 months in 45% of the cases) [62]. The median PFS for all bone sarcoma patients was 3.7 months (95% IC 3.4–4). There are ongoing biological studies associated with this trial, including the tumour microenvironment and genomic analyses of pre- and post-treatment tumour samples. These will be very informative in characterising the features of responsive tumours and the nature of the immune response.

A retrospective analysis of patients with relapsed, metastatic, or unresectable sarcomas treated with nivolumab and the multi-RTK inhibitor pazopanib (VEGFR, PDGFR, and KIT) further showed a CBR in two out of the three included osteosarcoma patients, of which one patient had a PR [65]. As with most other multi-RTK inhibitors trialled in paediatric and AYA sarcoma, pazopanib was also shown to alter the TME in a favourable way, by depleting immunosuppressive MDSCs and T reg cells and enriching activated T cells and cytotoxic NK effectors [66]. SD to this combination was also reported in three other sarcoma subtypes of AYA age, including one out of two SS patients, one MPNST patient, and one ASPS patient. The sole RMS and DSRCT patients included in this study both had a progressive disease. In a number of adult sarcoma subtypes treated with this same combination, a CBR was observed in 53% (8 out of 15 patients), which included dedifferentiated chondrosarcoma (1 PR and 1 SD out of 2 patients), epithelioid sarcoma (1 out of 2 PR), intimal sarcoma (1 out of 1 SD), and leiomyosarcoma (3 out of 7) subtypes [65]. The immunohistochemical evaluation revealed that the highest expression of PD-L1 was observed in the patients showing PR, although it was not a universal biomarker. Similar conclusions have been drawn from other studies, and reliable biomarkers to predict the response to combined-ICI-and-RTK-inhibitor therapy remain elusive in sarcomas.

## 7. Perspectives—Designing the Best ICI and RTKi Combination Strategy for Paediatric and AYA Sarcoma

Although the combined RTKi and anti-PD-1 clinical studies performed in paediatric and AYA sarcoma patients so far do point towards a clinical benefit in a subset of these patients, there is still considerable room to refine the drug combinations to maximise clinical benefit. In addition to performing more research on pinpointing the most appropriate response biomarkers, selecting the best RTKi to use in this combination, and identifying ICIs other than anti-PD-1/CTLA-4, is anticipated to lead to even better efficacy. Although there is currently little data available on the paediatric use of ICIs other than PD-1 or CTLA-4 inhibition, much more clinical data is available on RTKis in this regard. It is predictable that the use of an inhibitor specifically directed against RTKs on which a particular sarcoma subtype or individual patient’s tumour is dependent would be the most rational choice. If that RTKi also has immunomodulating properties, then this may present an effective “personalised” or tumour-specific combination with PD-1-directed therapy. This rationale is supported by an increasing body of evidence showing the success of several multi-RTKis and PD-1/PD-L1 inhibition in selected AYA and adult sarcoma subtypes.

The most profound clinical efficacy of RTK inhibitors combined with PD-1-targeted therapy has been observed in patients with ASPS and angiosarcoma (AS) STS subtypes [64]. From a molecular perspective, it is well-known that the VEGFRs are crucial drivers in these sarcoma subtypes [64,74], and as VEGFR is one of the most potently inhibited targets by the multi-RTKis tested in combination with ICIs, it makes sense that these tumours benefit from this particular combination. This is illustrated in the following clinical studies. In a heterogeneous group of STS patients, the combination of the PD-L1 antibody durvalumab and pazopanib showed encouraging efficacy with CR and PR observed in 1 and 12 out of 46 (ORR: 28%) evaluable patients [69]. In ASPS tumours specifically, however, the clinical success of axitinib plus pembrolizumab showed superior results. In addition to a CBR of 73%, objective responses were reported in 55% of patients, versus an ORR and CBR of 10% and 24%, respectively, in the non-ASPS sarcomas included in the same study. This suggests that similar improvements in paediatric sarcomas could be anticipated by selecting inhibitors that target the biologically important RTKs driving specific paediatric sarcoma progression [64]. Likewise, two out of three ASPS and one out of two AS obtained a partial response to the combination of nivolumab and sunitinib [63], and significant efficacy of camrelizumab and anlotinib was reported in an ASPS case report, with CR and PR noted in various lesions of the 24-year-old patient [67]. In this light, testing combinations of cabozantinib, anlotinib, or lenvatinib, which in addition to VEGFRs also target MET, PDGFR, and/or KIT, and have shown significant clinical efficacy in the majority of included osteosarcomas and Ewing sarcoma patients as a monotherapy, would be a logical way forward for testing in these paediatric sarcomas with ICIs. This is further underlined by a favourable initial tumour response observed in a 20-year-old stage-4 ASPS patient when treated with the combination of cabozantinib and nivolumab. As is characteristic for ASPS, this patient harboured the pathognomonic ASPL-TFE3 translocation, which is known to activate MET. Despite having only received 13 days of daily cabozantinib and one dose of nivolumab due to febrile neutropenia, three large external masses dramatically decreased in size with necrosis in two. Although a dose reduction was required to manage the side effects, the dual VEGFR- and MET-targeting properties of cabozantinib make this drug of particular interest in this disease. It further underlines a rationale for target-based selection of the RTK inhibitor most likely to achieve an anti-tumour effect in a particular sarcoma, either alone or in combination with ICI, while closely monitoring toxicity [68].

Another lesson learned from adult sarcomas is that when combining a particular RTKi with ICI, it is important to assess the immunomodulatory potential of the selected RTKi. In advanced/unresectable and kinase-refractory gastro-intestinal stromal tumours (GIST) and other advanced sarcoma patients, for example, combination of the TKI dasatinib (Src-family kinases (SFKs), KIT, ABL) with ipilimumab had no synergistic effects, and no objective responses were observed [73]. Although by Choi-criteria, which takes not only the size of the tumour but also drug-induced necrosis into account [75], a CBR of 77% and 60% for the 13 evaluable GIST and 5 evaluable non-GIST sarcoma patients was reported; the overall conclusion was that this combination in this type of cancer has only limited efficacy. Moreover, dasatinib did not alter the immune microenvironment favourably to enhance responses with the checkpoint inhibitor ipilimumab. The possible implication is that other RTKis, which do have immunomodulatory effects, in combination with PD-1-targeted therapies, would be a more promising way forward [73]. A recent interim report on the efficacy of the VEGFR-inhibitor cediranib and durvalumab in adult soft tissue sarcoma further showed a decrease in the tumour growth rate in 6 out of 10 evaluable leiomyosarcoma patients, which translated to stable disease in a small fraction of these patients. Leiomyosarcomas are usually considered “cold” tumours, and it was hypothesized that this combination would increase immunogenicity by recruiting tumour-infiltrating lymphocytes. However, in this study, cediranib could not drive a conversion from a cold-to-hot immunophenotype, which is supported by other reports showing that cediranib does not have favourable effects on the tumour immune microenvironment, and this might explain why, at best, SD was observed in only a small number of leiomyosarcoma patients [76,77,78]. This nicely emphasises the critical importance of integrating a deep and comprehensive understanding of the kinases driving tumour biology and those regulating the TME and of using this biological understanding to advance the combination of ICI and RTKi.

## 8. Conclusions

Immunotherapies harness a patient’s own immune system to recognise and kill cancer cells and offer new hope for the treatment of children with sarcomas that are resistant to standard therapies. Although ICIs have shown some potential in sarcoma, monotherapy approaches are not effective in most patients. To extend the benefits of immunotherapy to more patients with sarcoma, we propose that combination therapy strategies can be devised based on both the molecular and immune profiles of patients. This would bring together RTKis, which both modulate the TME and target kinases on which sarcoma subtypes have a clear dependency, with ICI therapies currently in clinical trials in several sarcoma subtypes, including paediatric sarcomas. Early clinical data in paediatric sarcomas show the potential of such combinations. As newer RTKis, such as lenvatinib and cabozantinib, have shown greater clinical efficacy in paediatric sarcomas and immunomodulating effects, combination of these agents with PD-1 inhibition is expected to further increase their efficacy. Implementation of combined and appropriate RTK- and ICI-specific biomarkers is expected to further refine the group of paediatric and AYA sarcoma patients most likely to respond to these combinations. Altogether, we propose that selection of the right RTKi and ICI based on a patient’s molecular profile, TME and/or sarcoma subtype-specific biology will pave the road towards achieving meaningful clinical activity in paediatric and AYA sarcoma patients.

## Figures and Tables

**Table 1:** Immunomodulatory effects of receptor tyrosine kinase inhibitors on the tumour immune microenvironment.

Inhibitor	Immunomodulatory Effect on Immunosuppressive Cells	References
Apatinib	Decreased the number of MDSCs and TAMs and increased the numbers of CD8^+^ T cells in mouse model of lung cancer	[53]
Anlotinib	Decreased the number of M2 TAMs and increased numbers of NK cells, dendritic cells, and M1 TAMs in mouse model of lung cancer	[54]
Axitinib	Decreased the number of MDSCs, TAMs, and regulatory T cells in mouse model of colon cancer and lung cancer	[55]
Cabozantinib	Decreased the number and function of MDSCs in a mouse model of prostate cancer	[48]
Lenvatinib	Decreased the number of TAMs in a mouse model of liver cancer and thyroid cancer Decreased TAMs and increased T cells in a mouse model of colon cancer	[51,56] [57]
Regorafenib	Decreased TAMs in a mouse model of colon cancer	[58]
Sunitinib	Decreased MDSCs and increased the number of CD8^+^ T cells in mouse model of kidney cancer	[59]

MDSCs: myeloid-derived suppressor cells; TAMs: tumour-associated macrophages; NK: natural killer.

**Table 2:** Anti-PD-1 in combination with receptor tyrosine kinase inhibitors in pre-clinical studies and clinical trials for adult and paediatric cancers.

Inhibitor	Pre-Clinical Studies	Clinical Trials
Apatinib	Potentiates anti-PD-1 in mouse model of lung cancer [53]	Biliary tract, cholangiocarcinoma (NCT04834674, NCT04720131) Cervical cancer (NCT03816553, NCT04974944) CRC (NCT03912857) Gastric Cancer (NCT03954756, NCT03878472, NCT04006821, NCT04267549, NCT04948125) HCC (NCT03839550, NCT03793725, NCT04014101, NCT03722875, NCT04826406, NCT04985136) Liver cancer (NCT03092895) Melanoma (NCT03955354) NSCLC (NCT03777124) Oesophageal cancer (NCT03736863, NCT03603756) Ovarian cancer (NCT04068974, NCT04507750) SCLC (NCT03417895) **Sarcomas (NCT04239443)** **Sarcoma, including paediatric/AYA (NCT03711279, NCT04126993, NCT03359018, NCT04074564, NCT04447274)** Solid tumours (NCT03491631) TNBC (NCT03945604, NCT03394287)
Anlotinib	Potentiates anti-PD-1 in mouse model of lung cancer [54]	HCC (NCT04052152) NSCLC (NCT03765775, NCT04507906, NCT04670107) SCLC (NCT04055792) **Soft tissue sarcomas, including AYA (NCT03946943)** **Soft tissue sarcoma (NCT04172805)**
Axitinib	Potentiates anti-PD-1 in a mouse model of colon cancer and lung cancer [55]	Melanoma (NCT04493203) RCC (NCT03595124, NCT02853331, NCT03172754 **Soft tissue sarcomas, including AYA (NCT02636725)**
Cabozantinib	Potentiates anti-PD-1 in a mouse model of prostate cancer [48]	Endometrial cancer (NCT03367741) Genitourinary tumours (NCT03866382, NCT02496208) HCC (NCT01658878, NCT03299946) RCC (NCT03729245, NCT03793166, NCT03937219, NCT03635892, NCT03141177) Thyroid cancer (NCT03914300) TNBC (NCT03316586) **Soft tissue sarcoma (NCT04551430, NCT04339738, NCT04149275)** Solid tumours (NCT04514484)
Lenvatinib	Potentiates anti-PD-1 in mouse models of liver cancer [51] thyroid cancer [56] and colon cancer [57]	Endometrial cancers (NCT03517449) Gastroesophageal cancer (NCT03321630) Liver cancers (NCT03895970, NCT03779100, NCT03951597, NCT04042805, NCT03713593, NCT04997850) Melanoma (NCT03820986) NSCLC (NCT03976375, NCT03829332, NCT03829319, NCT03516981) **Advanced sarcoma (NCT04784247)** Solid tumours (NCT03797326) TNBC (NCT04427293) Urothelial cancers (NCT03898180)
Regorafenib	Potentiates anti-PD-1 in mouse models of colon cancer [58]	CRC (NCT03657641) Gastroeosophageal cancer (NCT04879368) HCC (NCT03347292, NCT04183088, NCT04170556, NCT04310709, NCT04696055) **Osteosarcoma, including paediatric (NCT04803877)**
Sunitinib	Potentiates anti-PD-1 in mouse model of kidney cancer [59]	**Sarcomas, including paediatric (NCT03277924)** RCC (NCT03729245, NCT02960906, NCT03075423)

**In bold**: trial focussed on sarcoma patients; **bold-underlined**: trial including paediatric and/or AYA sarcoma patients. CRC: colorectal cancer; GIST: gastro-intestinal stromal tumours; HCC: hepatocellular carcinoma; NSCLC: non-small cell lung cancer; PD-1, programmed death-1; RCC: renal cell carcinoma; SCLC: small cell lung cancer; TNBC: triple-negative breast cancer.

**Table 3:** Clinical results and ongoing trials on combined RTKi and ICI therapy in sarcoma.

Study/Trial	Drugs	Key Targets	Inclusion	Age	Results	Status (September 2021)
**Apatinib**						
NCT04126993	Apatinib + camrelizumab (SHR-1210)	Mainly VEGFR2 PD-1	Sarcoma	14–75 years	NA	Unknown
NCT03711279	Apatinib + camrelizumab (SHR-1210)	Mainly VEGFR2 PD-1	STS	16–70 years	NA	Recruiting
NCT03359018	Apatinib + camrelizumab (SHR-1210)	Mainly VEGFR2 PD-1	Advanced osteosarcoma	11 years +	*Osteosarcoma* [60] CR or PR: 9/43 OS patients ORR: 21%; CBR: 30%	Completed
NCT04239443	Apatinib + SHR-1210	Mainly VEGFR2 PD-1	Advanced NSCLC, STS, uterine cancer	18–70 years	NA	Recruiting
NCT04074564	Apatinib + PD1 antibody (unspecified) + Multi-antigen autoimmune cell injection (MASCT-I)	Mainly VEGFR2 PD-1 Adoptive cellular immunotherapy	Advanced osteosarcoma and STS	14–70 years	NA	Not yet recruiting
NCT04447274	Apatinib + carilizumab	Mainly VEGFR2 PD-1	Unresectable UPS and ASPS	16 years +	NA	Not yet recruiting
**Anlotinib**						
NCT03946943	Anlotinib + toripalimab	VEGFR, PDGFR, FGFR, KIT PD-1	Advanced UPS	16 years +	NA	Not yet recruiting
NCT04172805	Anlotinib + toripalimab	VEGFR, PDGFR, FGFR, KIT PD-1	Advanced STS	18–70 years	NA	Recruiting
Case report	Anlotinib + camrelizumab	VEGFR, PDGFR, FGFR, KIT PD-1	ASPS	24 years	ASPS (*n =* 1): significant efficacy, CR and PR in various lesions [67]	NA
**Axitinib**						
NCT02636725	Axitinib pembrolizumab +	Mainly VEGFR PD-1	Advanced ASPS and other STS	16 years +	*ASPS (n =* 11) [64] PR: 6/11; SD: 2/11 ORR 55%; CBR: 73% *Non-ASPS STS (n =* 21) PR (2/21): 1/1 EPI, 1/2 stLMS MR * (3/21): 1/2 stLMS, 1/1 SynS, 1/5 HGP ORR: 10%; CBR: 24%	Active, not recruiting
**Cabozantinib**						
NCT04551430	Cabozantinib + nivolumab + ipilimumab	VEGFR2, MET, AXL PD1 CTLA-4	Metastatic STS	18 years +	NA	Recruiting
NCT04149275	Cabozantinib + nivolumab + ipilumumab	VEGFR2, MET, AXL PD1 CTLA-4	Recurrent gynaecologic carcinosarcoma	18 years +	NA	Withdrawn (funding)
NCT04339738	Cabozantinib or paclitaxel + nivolumab	VEGFR2, MET, AXL or chemo PD1	Advanced STS (mainly AS)	18 years +	NA	Recruiting
Case report	Cabozantinib + nivolumab	VEGFR2, MET, AXL PD1	ASPS	20 years	ASPS (*n* = 1): Significant reduction in tumour size [68]	NA
**Lenvatinib**						
NCT04784247	Lenvatinib + pembrolizumab	VEGFR, PDGFR, FGFR, KIT PD-1	Advanced sarcoma	18 years +	NA	Recruiting
**Regorafenib**						
NCT04803877	Regorafenib + nivolumab	VEGFR, PDGFR, FGFR, KIT PD-1	Osteosarcoma (SARC038)	5 years +	NA	Recruiting
**Sunitinib**						
NCT03277924	Sunitinib + nivolumab	VEGFR2, PDGFRB, KIT PD-1	Advanced STS and bone sarcoma	12–80 years	*Bone sarcoma cohort* (*n* = 40) [62] CR: 1/14 chondrosarcoma PR: 1/17 osteosarcoma SD: 22 not further specified bone sarcoma patients ORR: 5%; CBR: 60% *STS cohort* (*n* = 59) [63] Phase 1b (*n* = 13): PR (6/13): 2/4 CSS, 2/3 ASPS, 1/2 SynS, 1/2 AS SD: 3/13 not further specified Phase 2 (*n* = 46): CR (1/46): 1/5 AS PR (5/46): 2/4 ASPS, 1/5 AS, 1/4 emCS, 1/9 SynS SD: 33/46 not further specified STS overall ORR: 20%; CBR: 81%	Recruiting
**Pazopanib**						
NCT03798106	Pazopanib + durvalumab	VEGFR, PDGFR, FGFR, KIT PD-L1	Metastatic STS	19 years +	STS: 1/46 CR and 12/46 PR [69] ORR: 28%	Recruiting
Retrospective study	Pazopanib + nivolumab	VEGFR, PDGFR, FGFR, KIT PD-1	Advanced sarcoma	24–78 years	*Paediatric/AYA subtypes* (*n* = 9) [65] OS: 1/3 PR, 1/3 SD, 1/3 PD SynS: 1/2 SD, 1/2 PD MPNST: 1/1 SD ASPS: 1/1 SD RMS: 1/1 PD DSRCT: 1/1 PD ORR: 11%; CBR: 56% *Adult type sarcomas* (*n* = 15) DC: 1/2 PR, 1/2 SD EPS: 1/2 PR, 1/2 PD IS: 1/1 SD LMS: 3/7 SD; 4/7 PD PD: 1/1 mCS, 1/1 LPS, 1/1 UPS ORR: 13%; CBR: 47%	NA
Case report	Pazopanib + pembrolizumab	VEGFR, PDGFR, FGFR, KIT PD-1	Advanced undifferentiated UPS	63 years	UPS (*n* = 1): disease regression for minimum of 10 months [70]	NA
**Other RTK- and TK-associated inhibitors (with anti-angiogenic component)**						
NCT04579757	Surufatinib + tislelizumab	VEGFR1-3, FGFR1, CSF-1R PD-1	Advanced solid tumours (incl STS)	18 years +	NA	Recruiting
NCT04044378	Famitinib + camrelizumab	VEGFR, PDGFR, KIT PD-1	Advanced osteosarcoma	12 years +	NA	Withdrawn (toxicity)
NCT03919539	Famitinib + camrelizumab (EBAOFC)	VEGFR, PDGFR, KIT PD-1	Advanced osteosarcoma	12 years +	NA Epigenetic biomarker study	Recruiting
NCT02298959	ZIV-aflibercept pembrolizumab +	VEGFA/B PD-1	Advanced solid tumours (incl sarcoma)	18 years +	*Various sarcoma* (*n* = 11) [71] SD (7/11): 2/2 LPS, 1/1 EWS, 1/1 chordoma, 1/1 uLMS, 1/1 GIST, 1/1 HGP ORR: 0%; CBR: 64%	Recruiting
NCT03141684	Bevacizumab atezolizumab +	VEGFA PD-L1	Advanced ASPS	2 years +	NA	Recruiting
**Other RTK and TK inhibitors (without anti-angiogenic component)**						
NCT03609424	Imatinib + spartalizumab (PDR-001)	PDGFR, KIT, ABL PD-1	Metastatic or unresectable GIST	18 years +	NA	Recruiting
NCT04242238	DCC-3014 + avelumab	CSF-1R PD-L1	Advanced or metastatic sarcoma	18 years +	*LMS (n* = 7) [72] SD: 2/7 ORR 0%; CBR: 29% *Non-LMS (n* = 6) SD: 1/6 not further specified ORR: 0%; CBR: 17%	Recruiting
NCT01643278	Dasatinib + ipilimumab	SFKs, KIT, ABL CTLA4	Refractory GIST and other advanced sarcomas	18 years +	*GIST* (*n* = 13) [73] No response by RECIST criteria Choi criteria PR: 7/13; SD: 3/13 ORR: 54%; 77% CBR *Non-GIST (n* = 5) RECIST SD (3/5): 1/2 HGs, 1/1 CSS, 1/1 EPI ORR: 0%; 60% CBR	Completed

* MR: minor response (decrease in size of target lesion of less than 30%). CBR: clinical benefit rate; CR: complete response; ORR: objective response rate; PD: progressive disease; PR: partial response. AS: angiosarcoma; ASPS; alveolar soft part sarcoma; CSS: clear cell sarcoma; emCS: extraskeletal myxoid chondrosarcoma; mCS: mesenchymal CS; EPI: epithelioid sarcoma; EWS: Ewing sarcoma, GIST: gastro-intestinal stromal tumour; HGP: high-grade pleomorphic sarcoma; HGs: high-grade sarcoma; OS: osteosarcoma; stLMS: soft tissue leiomyosarcoma (non-uterine LMS); uLMS: uterine LMS; LPS: liposarcoma; STS: soft tissue sarcoma; SynS: synovial sarcoma; UPS: undifferentiated pleomorphic sarcoma. NA: not applicable. Responses are by RECIST criteria (best overall response) unless otherwise specified.

## Data Availability

Not applicable.
